# Only rare classical MHC-I alleles are highly expressed in the European house sparrow

**DOI:** 10.1098/rspb.2023.2857

**Published:** 2024-02-21

**Authors:** Hannah Watson, Anna Drews, Kelly Hultman Skogsmyr, Júlio Manuel Neto, Jacob Roved, Helena Westerdahl

**Affiliations:** ^1^ Department of Biology, Lund University, 223 62 Lund, Sweden; ^2^ Department of Biology and Centre for Environmental and Marine Studies, University of Aveiro, Campus de Santiago, 3810-193 Aveiro, Portugal; ^3^ Section for Molecular Ecology and Evolution, GLOBE Institute, University of Copenhagen, Øster Farimagsgade 5, Bygn. 7, 1353 Copenhagen K, Denmark

**Keywords:** major histocompatibility complex, adaptive immunity, pathogen-mediated selection, immune genes, pathogens

## Abstract

The exceptional polymorphism observed within genes of the major histocompatibility complex (MHC), a core component of the vertebrate immune system, has long fascinated biologists. The highly polymorphic *classical* MHC class-I (MHC-I) genes are maintained by pathogen-mediated balancing selection (PMBS), as shown by many sites subject to positive selection, while the more monomorphic *non-classical* MHC-I genes show signatures of purifying selection. In line with PMBS, at any point in time, rare classical MHC alleles are more likely than common classical MHC alleles to confer a selective advantage in host–pathogen interactions. Combining genomic and expression data from the blood of wild house sparrows *Passer domesticus*, we found that only rare classical MHC-I alleles were highly expressed, while common classical MHC-I alleles were lowly expressed or not expressed. Moreover, highly expressed rare classical MHC-I alleles had more positively selected sites, indicating exposure to stronger PMBS, compared with lowly expressed classical alleles. As predicted, the level of expression was unrelated to allele frequency in the monomorphic non-classical MHC-I alleles. Going beyond previous studies, we offer a fine-scale view of selection on classical MHC-I genes in a wild population by revealing differences in the strength of PMBS according to allele frequency and expression level.

## 1. Introduction

Understanding the mechanisms by which genetic variation is maintained in natural populations remains a fundamental question in evolutionary biology. *Classical* genes of the major histocompatibility complex (MHC) are among the most polymorphic genes in vertebrate genomes and play a central role in adaptive immunity [1–3]. The maintenance of high polymorphism across paralogous genes in the MHC genomic region has long been of intense interest among ecologists and evolutionary biologists (reviewed in [4,5]). Accumulating evidence confirms that the exceptional polymorphism at the MHC is maintained by pathogen-mediated balancing selection (PMBS), mediated by negative frequency-dependent selection, heterozygote advantage and/or fluctuating selection [4,5].

Under negative frequency-dependent selection, hosts are conferred a disadvantage when carrying common alleles to which the pathogens have had time to adapt, while rare or novel alleles are often associated with a selective advantage [6]. The theory of heterozygote advantage states that individuals with high heterozygosity, and thus higher allelic diversity, are more likely to successfully present antigens and elicit an adaptive immune response to a diverse range of pathogens, compared with homozygous individuals [7]. Since rare alleles are more likely to occur at heterozygous loci, while common alleles are more likely to occur at homozygous loci, heterozygote advantage can potentially promote rare alleles and disfavour common alleles, similar to negative frequency-dependent selection. Consistent with PMBS theory, many studies have found high rates of positive selection in classical MHC genes (reviewed in [8,9]) and correlations between MHC genotype and resistance to infection [10–13]. Moreover, studies in humans have reported direct mechanistic links between MHC antigen presentation and disease resistance [14]. Nevertheless, most previous studies have inferred historic PMBS on MHC genes from genetic footprints, while there is a lack of evidence for PMBS on more recent time scales in natural populations.

Along with the highly polymorphic *classical* MHC genes (in humans, named human leucocyte antigens, HLA-A, -B and -C), the MHC region is home to *non-classical* genes (the equivalent of HLA-E, -F and -G, in humans). While classical MHC genes exhibit high polymorphism, high gene expression and present peptides to T-cells, non-classical MHC genes are characterized by the absence of one or more of the key features of classical genes [15]. Non-classical MHC molecules can have a wide range of functions, which can be immune- or non–immune-related [16,17]. While PMBS is expected to maintain high polymorphism in classical MHC genes, purifying selection is thought to be the main selective force acting on the more monomorphic non-classical MHC genes. Even though non-classical genes have been reported in many vertebrate taxa [15,18–20], there has been limited effort to distinguish between classical and non-classical genes in evolutionary and ecological studies, thus overlooking their different functions in immunity.

To date, the ecological and evolutionary literature has emphasized the importance of individual MHC diversity in genomic DNA (total number of unique MHC alleles per individual) in the ability to fight a large range of pathogens, whereas the role of gene expression has largely been neglected [21,22]. Ultimately, it is the assemblage of different MHC molecules expressed on the cell surface, rather than the variation in the genome, as well as the level at which MHC variants are expressed, that determines an individual's capability to trigger an adaptive immune response to an invading pathogen [23]. Moreover, higher levels of MHC expression (transcription) are associated with a more robust immune response [24], and quantifying gene expression is therefore central to the reliable characterization of an individual's putative MHC phenotype in species with high MHC diversity. Recently, transcribed MHC sequences (mRNA) have been quantified as a measure of expressed diversity in wild songbirds, revealing that only a proportion of MHC genes are expressed and the proportion of expressed alleles is more similar within, than between, species [25,26]. In a study of three congeneric songbird species belonging to the true sparrows (*Passer* spp.), MHC genomic diversity was found to be highly variable between species, yet expressed MHC diversity was highly conserved among individuals [27]. A reliance on measures of individual genomic MHC-I diversity may thus overestimate functional MHC-I diversity.

Among vertebrates, songbirds stand out as exhibiting extraordinarily high MHC diversity, not only compared with humans and non-human mammals but also compared with other bird orders [22,28]. Non-classical MHC genes are most likely common among songbirds, but they have so far been challenging to separate from classical genes, limiting the possibilities for investigating recent selection on the two gene types. In songbirds belonging to the true sparrows, non-classical MHC class I (MHC-I) genes can be separated from classical MHC-I genes on the basis of a 6 bp deletion [18,27], thus offering excellent study systems to investigate recent selection at the MHC. Earlier work in sparrows has shown considerable variation in the number of sites subject to positive selection between classical and non-classical MHC-I genes; classical MHC-I genes have an antigen-binding region with many sites subject to positive selection, consistent with balancing selection, whereas non-classical genes have few, or even no, sites subject to positive selection, in agreement with purifying selection [18,29,30].

Here, we set out to test whether recent selection at the MHC echoes historic selection in a natural population of the European house sparrow *Passer domesticus*. We study recent selection by quantifying allele frequencies and gene expression in classical and non-classical MHC-I genes, using blood samples collected from a wild population. Theory predicts that PMBS—via one or more mechanisms—confers a selective disadvantage to classical MHC-I alleles when they are common, while selection is more likely to favour rare alleles. On the contrary, non-classical alleles, which are thought to be subject to purifying selection, are not expected to show any selective advantage or disadvantage in relation to allele frequency. We thus hypothesize that recent PMBS maintains more rare classical MHC-I alleles in the population, compared with non-classical alleles, which are expected to be less diverse and more commonly shared among individuals as a result of purifying selection. Lastly, we explore how the degree of MHC-I allele expression varies in relation to allele frequency and genetic footprints of historic selection.

## 2. Results

### (a) Low genomic diversity, but high expressed diversity, among classical alleles

Among 28 European house sparrows that were genotyped for MHC-I, at both the genomic (gDNA) and expression (cDNA) levels from blood, 133 unique genomic nucleotide alleles were identified, separating into 68 classical and 65 non-classical alleles. At the population level, 50 classical and 20 non-classical nucleotide alleles were expressed, hence a large proportion of classical alleles was expressed (73.5%) in at least one individual, while a smaller subset of non-classical alleles was expressed (30.8%; table 1).

**Table 1.** Summary of diversity in genomic and expressed MHC-I alleles in the house sparrow at the level of the population (total counts of genomic and expressed alleles and percentage expressed) and individual (average counts of genomic and expressed alleles and percentage expressed per individual). Data are presented for both nucleotide and amino acid alleles and classical and non-classical alleles. Individual allele counts are presented as the mean ± s.e.m. of model-fitted values (see table 2 and electronic supplementary material, table S1).

		nucleotide		amino acid	
		classical	non-classical	classical	non-classical
genomic	population	68	65	62	39
	individual	5.47 ± 0.46	7.28 ± 0.54	5.29 ± 0.43	5.96 ± 0.46
expressed	population	50 (73.5%)	20 (30.8%)	47 (75.8%)	9 (23.1%)
	individual	3.21 ± 0.36	2.37 ± 0.30	3.02 ± 0.33	2.07 ± 0.27
		(57.2 ± 2.4%)	(37.9 ± 2.5%)	(58.6 ± 2.3%)	(38.6 ± 2.8%)

At the individual level, sparrows exhibited lower MHC-I genomic diversity among classical alleles, compared with non-classical alleles (table 1; table 2, model *a*; *p* = 0.0070). The number of expressed alleles per individual was positively correlated with the number of genomic alleles (table 2, model *b*; *p* = 0.045), but the slope of the relationship was less than 1, indicating that—in line with previous studies—only a fraction of alleles were expressed. Although sparrows carried more non-classical alleles, compared with classical alleles, this did not lead to the expression of more non-classical alleles. In fact, there was a tendency towards the expression of fewer non-classical, compared with classical, alleles (table 1; table 2, model *b*; *p* = 0.082). When considering amino acid alleles, genomic diversity did not differ between classical and non-classical alleles, but—consistent with findings from nucleotide alleles—expressed diversity was lower among non-classical alleles (table 1; electronic supplementary material, table S1, models *a* and *b*). Genomic and expressed MHC-I diversity did not differ between spring and autumn (table 2; all *p* ≥ 0.54), and little variance was explained by the sampling site (table 2, models *a* and *b*). Thus, for the purposes of this study and from here on, we refer to a single population.

**Table 2.** Summary of statistical analyses investigating variation in (*a*) genomic and (*b*) expressed diversity, (*c* and *d*) allele distribution frequencies, and (*e* and *f*) expression patterns between classical and non-classical MHC-I alleles in the house sparrow. Fixed and random effects are shown along with parameter estimates (mean ± s.e.m.), chi-square statistics (from likelihood ratio tests or Wald tests in the case of model *d*) and associated *p*-values. Fixed effects dropped from models are shown in italics.

model and parameters	*β* ± s.e.m.	*χ*^2^	*p*-value
(*a*) individual MHC-I genomic diversity—nucleotide alleles			
allele type (non-classical)	0.29 ± 0.11	7.27	0.0070
*season* (*spring*)	−*0.071 ± 0.12*	*0*.*37*	*0*.*54*
random: site/identity	1.16 × 10^−2^ ± 0.11		
(*b*) individual MHC-I expressed diversity—nucleotide alleles			
number of gDNA alleles	0.066 ± 0.033	4.022	0.045
*allele type* (*non-classical*)	*−0.30 ± 0.18*	*3*.*018*	*0*.*082*
*season* (*spring*)	−*0.032 ± 0.17*	*0*.*036*	*0*.*85*
*number of gDNA alleles : allele type*	−0.012 *± 0.073*	*0*.*025*	*0*.*87*
random: site/bird ID	1.16 × 10^−10^ ± 1.080 × 10^−5^		
(*c*) MHC-I genomic allele count in relation to allele frequency—nucleotide alleles			
allele type (non-classical)	−0.64 ± 0.30		
allele frequency	−0.54 ± 0.078		
allele type (non-classical) : allele frequency	0.22 ± 0.094	5.57	0.018
(*d*) MHC-I genomic allele count in relation to allele frequency—amino acid alleles			
allele type (non-classical)	−1.44 ± 0.34		
allele frequency	−0.52 ± 0.075		
allele type (non-classical) : allele frequency	0.34 ± 0.088	16.00	<0.001
(*e*) MHC-I expression in relation to allele frequency—nucleotide alleles			
expression probability: zero count model (logit)			
allele type (non-classical)	2.30 ± 0.40		
allele frequency	0.33 ± 0.073		
number of gDNA alleles	0.061 ± 0.029	4.34	0.037
allele type : allele frequency	−0.36 ± 0.080	20.20	<0.001
* season* (*spring*)	*0.071 ± 0.25*	*0*.*085*	*0*.*77*
random: site/bird ID	2.81 × 10^−9^ ± 5.30 × 10^−5^		
expression level: count model (log)			
allele type (non-classical)	−1.50 ± 0.24		
allele frequency	−0.40 ± 0.042		
log cDNA read depth	1.14 ± 0.35	10.41	0.0013
number of cDNA alleles	−0.13 ± 0.047	7.81	0.0052
allele type : allele frequency	0.38 ± 0.049	60.12	<0.001
* season* (*spring*)	−*0.11 ± 0.15*	*0*.*52*	*0*.*47*
random: site/bird ID	1.08 × 10^−9^ ± 3.29 × 10^−5^		
(*f*) MHC-I expression within the individual genotype—nucleotide alleles			
allele rank^a^	−58.89		
allele type (non-classical)	−3.63 ± 2.72		
log cDNA read depth	27.47 ± 4.31	34.47	<0.001
*number of cDNA alleles*	−*0.75 ± 0.64*	*1*.*38*	*0*.*24*
allele rank : allele type (non-classical)^a^	53.42	152.35	<0.001
random: bird ID	1.94 × 10^−8^ ± 1.39 × 10^−4^		

^a^Parameter estimate is the sum of linear, quadratic, cubic and ^ˆ^4 parameters, and hence no standard error is given.

### (b) Most genomic MHC-I alleles are rare

**Figure 1.**
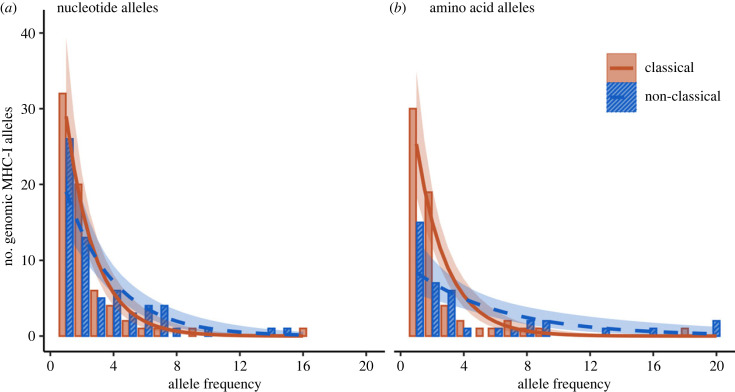
Relationship between MHC-I genomic allele count and allele frequency in a population of house sparrows among (*a*) nucleotide alleles and (*b*) amino acid alleles. Most genomic MHC-I alleles are rare: the number of genomic MHC-I alleles decreased rapidly as alleles increased in frequency within the population (i.e. were found in more individuals and thus more common), meaning most alleles occurred at low frequencies in the population, while few alleles were shared among many individuals. The pattern was more marked (i.e. a significantly more negative slope) for classical (orange, solid bars, solid line) alleles, compared with non-classical (blue, striped bars, dashed line) alleles, and the difference between classical and non-classical alleles was larger among amino acid alleles (*b*), compared with nucleotide alleles (*a*). Lines represent fitted means with 95% confidence intervals shown. Bars represent raw counts of unique alleles.

Since PMBS is expected to confer a disadvantage to hosts carrying common alleles, while rare or novel alleles are often associated with a selective advantage, we expect that more classical MHC-I alleles will be rare, compared with non-classical MHC-I alleles. Purifying selection does not confer any advantage or disadvantage based on the frequency of alleles in the population, and thus it is expected that non-classical MHC-I alleles are more commonly shared among individuals, particularly at the level of amino acid alleles. To test this hypothesis, we compared binned counts of classical versus non-classical MHC-I alleles according to the frequency of occurrence among the 28 house sparrows. Overall—accounting for both classical and non-classical alleles—the majority of the 133 genomic nucleotide MHC-I alleles were rare: 91 alleles (68.4%) occurred in only one or two individuals (i.e. low frequency; less than 8% of the population), while just six alleles occurred in eight or more individuals (with the highest allele frequency being 16). However, the shape of the curve describing the relationship between nucleotide allele count (i.e. number of unique alleles) and allele frequency (i.e. occurrence in population) varied between classical and non-classical alleles (table 2, model *c*; figure 1*a*; *p* = 0.018). The slope describing the relationship was more negative (i.e. steeper) among classical (estimated marginal (EM) trend = −0.54 [−0.70, −0.39]), compared with non-classical (EM trend = −0.33 [−0.43, −0.22]), alleles (figure 1*a*). The difference between the two allele types was even more pronounced for genomic amino acid alleles (table 2, model *d*; figure 1*b*; *p* < 0.001); the slope for classical amino acid alleles was similar to that for nucleotide alleles (EM trend = −0.52 [−0.67, −0.37]), yet the slope for non-classical amino acid alleles was less negative (EM trend = −0.18 [−0.27, −0.088]). In other words, classical alleles were shared infrequently among many individuals, while a considerable proportion of non-classical alleles were shared among several individuals.

Consistent with the findings of Drews *et al*. [27], phylogenetic analysis placed all non-classical MHC-I alleles in a distinct cluster with high bootstrap support, while low bootstrap support was observed for all basal nodes among classical MHC-I alleles (electronic supplementary material, figure S1). No clear phylogenetic signal was observed based on allele frequency, with rare and common classical MHC-I alleles distributed across the phylogenetic tree (electronic supplementary material, figure S1).

### (c) Rare classical MHC-I alleles are highly expressed

**Figure 2.**
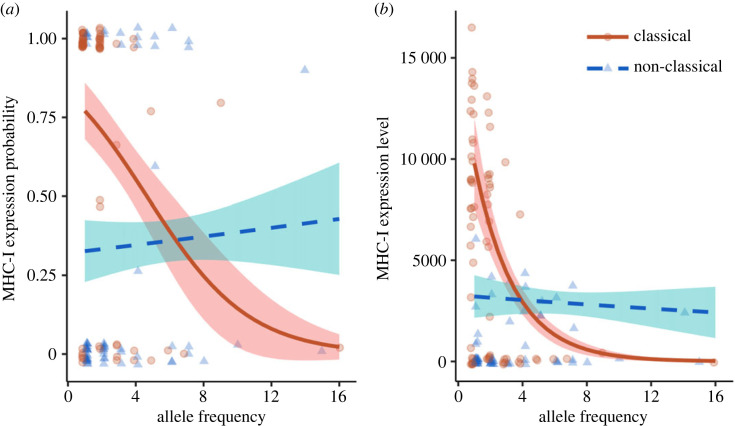
(*a*) Probability and (*b*) level of expression of MHC-I nucleotide alleles in relation to allele frequency in house sparrows. Classical (orange, circles, solid line) MHC-I alleles were more likely to be expressed and expressed at a higher level when rare, while the probability and level of expression of non-classical (blue, triangles, dashed line) MHC-I alleles were independent of allele frequency. Lines represent fitted means with 95% confidence intervals shown. Raw values in (*a*) represent the proportion of birds expressing each allele, while raw values in (*b*) represent the mean expression level for each allele.

Since theory predicts differences in selective advantage in relation to allele frequency between the two gene types, we subsequently explored if, and how, the degree of expression might be related to allele frequency. We found that both the probability of expression (table 2, model *e* [zero-count model]; figure 2*a*;) and level of expression (table 2, model *e* [count model]; figure 2*b*) varied with allele frequency, but contrasting patterns were observed between classical and non-classical nucleotide alleles (allele type : allele frequency: all *p* < 0.001). Rare classical MHC-I alleles (i.e. occurring at low frequencies, in just one or two individuals and less than 8% of the population) were more likely to be expressed (figure 2*a*; EM trend (1−logit) = −0.33 [−0.47, −0.19]) and were expressed at a higher level (figure 2*b*; EM trend (log) = −0.40 [−0.48, −0.32]) compared with classical alleles that were common within the population. By contrast, expression—both probability (figure 2*a*; EM trend (1−logit) = 0.029 [−0.038, 0.096]) and level (figure 2*b*; EM trend (log) = −0.019 [−0.069, 0.031])—of non-classical MHC-I alleles was independent of allele frequency as illustrated by 95% CIs overlapping with zero. When considering amino acid alleles, the relationships between expression and allele frequency were very similar to that observed among nucleotide alleles (electronic supplementary material, table S1, model *c*; electronic supplementary material, figure S2).

Expressed classical MHC-I nucleotide alleles had many sites (six) under positive selection, consistent with PMBS, while expressed non-classical alleles had no sites showing signs of positive selection (electronic supplementary material, table S2). The same pattern was also evident at the genomic level, where classical alleles had seven sites under positive selection, while non-classical alleles had between one and three positively selected sites, depending on the model used (electronic supplementary material, table S2), mirroring what has been shown in earlier studies of historic selection [27,29,30].

### (d) Sparrows have a single highly expressed classical MHC-I allele

**Figure 3.**
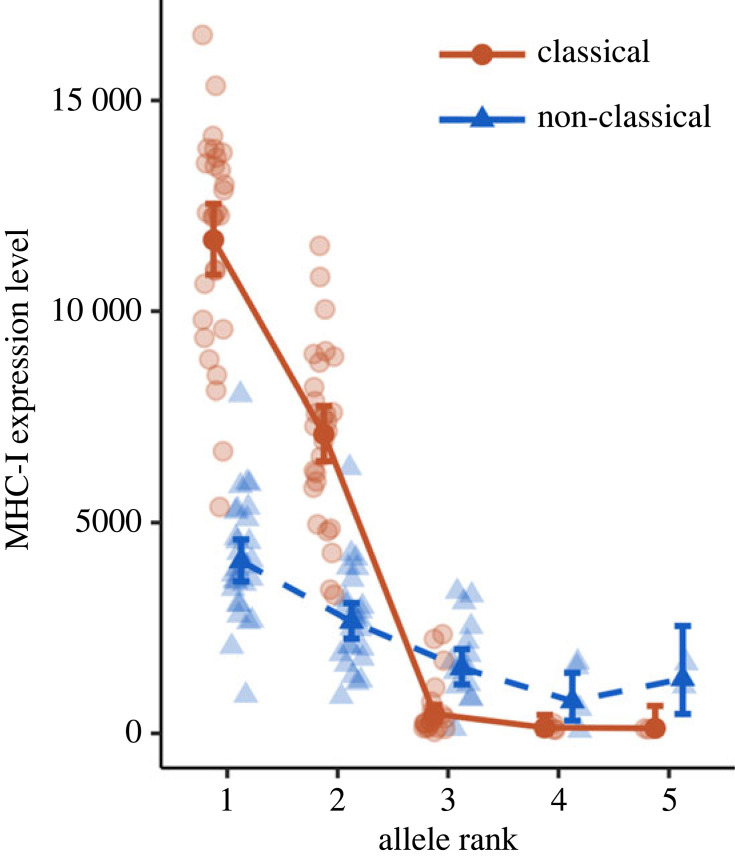
Individual expression levels of classical (orange, circles, solid line) and non-classical (blue, triangles, dashed line) MHC-I nucleotide alleles ranked in order of decreasing expression within an individual's genotype. On average (mean ± s.e.m.), house sparrows expressed 3.21 ± 0.36 (range: 2–5) classical, and 2.37 ± 0.30 (range: 1–5) non-classical, MHC-I nucleotide alleles. Plotted values represent model-fitted means with 95% confidence intervals, back-transformed from a square-root scale and overlaid on raw data observations.

Having observed large variation in expression levels among classical MHC-I alleles, yet little variation in expression among non-classical MHC-I alleles, we further investigated how expression patterns varied within each individual's genotype. We did this by analysing variation in expression levels between alleles within the genotype according to their rank order (ranked 1–5, from the highest to the lowest expression). Consistent with our previous findings, within an individual's MHC genotype, expression levels varied greatly between classical alleles, while there was less variation in expression among non-classical alleles (table 2, model *f*, figure 3; rank : allele type: *p* < 0.001). On average, sparrows had one highly expressed classical allele (rank 1) and one moderately expressed classical allele (rank 2; contrast between alleles ranked 1 and 2, and their respective contrasts with alleles ranked 3, 4 and 5: all *p* < 0.001), while the remaining classical alleles (ranks 3–5) were expressed at similarly low levels (all contrasts: *p* ≥ 0.45).

Expression levels differed significantly among the non-classical MHC-I alleles that were ranked 1, 2 and 3 (all contrasts: *p* ≤ 0.013), though the magnitude of differences was much smaller than among the first three ranked classical alleles. Absolute levels of expression of non-classical alleles never approached those of even the moderately (rank 2) expressed classical alleles (all contrasts: *p* < 0.001). Similar patterns were observed among amino acid alleles for both classical and non-classical alleles (electronic supplementary material, table S1, model *d*; electronic supplementary material, figure S3). Furthermore, the read depth of genomic DNA of the most highly expressed alleles suggests that some non-classical alleles are likely to be duplicated (electronic supplementary material, figure S4). Hence, it is likely that we underestimated the number of non-classical genes per individual and thereby overestimated the absolute levels of expression of the most highly expressed non-classical alleles (figure 3). There was no correlation between nucleotide allele read counts in cDNA and gDNA, confirming that there was no evidence for sequence bias due to e.g. primer specificity (Pearson's correlation: *R*^2^ = −0.012, *t* = −0.22, *p* = 0.83).

Among the classical MHC-I alleles, we estimated historic PMBS separately in the highly expressed, moderately expressed and lowly expressed alleles. This revealed that the highly (rank 1; *n* = 23) and moderately (rank 2; *n* = 24) expressed classical alleles had many sites (between seven and nine, depending on the model, albeit not the same sites in rank 1 and rank 2 alleles) under positive selection, consistent with strong PMBS, while lowly expressed classical alleles (ranks 3–5; *n* = 12) had few positively selected sites (three), suggesting they are subject to weaker PMBS (electronic supplementary material, table S2).

## 3. Discussion

Theory has long predicted that high polymorphism among classical MHC genes is maintained by PMBS, while low polymorphism observed among non-classical MHC genes is sustained by purifying selection. These opposing mechanisms of selection have been verified in terms of historic selection on the molecular level by the repeated finding that classical MHC genes have a high number of positively selected sites, while non-classical MHC genes have few or no positively selected sites [18,26,29,30]. However, until now, we have lacked evidence from natural populations indicating that more recent selection aligns with the two MHC-I gene types being subject to different selection mechanisms. Furthermore, not all classical alleles are expected to be equal, since PMBS should disfavour common alleles, yet no study has attempted to separate classical alleles by frequency or expression.

Here, in the European house sparrow, we inferred recent selection at the MHC using novel data of both MHC-I allele frequencies and gene expression levels. Our findings—that only rare classical MHC-I alleles are highly expressed whereas common classical MHC-I alleles are either lowly expressed or not expressed at all—support the theoretical disadvantage of common classical MHC alleles, associated with PMBS. We suggest that the highly expressed rare classical MHC-I alleles are subject to stronger balancing selection than the lowly expressed common classical alleles. This reasoning is supported by historic selection (i.e. the number of positively selected sites), with the finding that the highly and moderately expressed classical alleles have more than twice as many positively selected sites than the lowly expressed classical alleles. We thus clearly distinguish between two groups of classical alleles: the rare, highly or moderately expressed alleles, which—in both recent and historic times—have been subject to strong balancing selection, and the common, lowly expressed alleles that show evidence of being subject to weaker balancing selection.

In contrast to the findings in classical MHC-I alleles, we found that non-classical MHC-I alleles are less likely to be expressed and are, overall, lowly expressed compared with classical alleles. Moreover, the probability and level of expression of non-classical alleles are wholly independent of allele frequency. This is consistent with the theory that, unlike classical MHC-I alleles, non-classical MHC-I alleles are not subject to balancing selection. Furthermore, higher variance in expression levels, as observed among classical MHC-I alleles when compared with non-classical MHC-I alleles, is in line with ongoing balancing selection among classical MHC-I alleles [31,32]. The observed differences in allele frequencies and expression between classical and non-classical alleles are thus consistent with the two MHC-I gene types being subject to different mechanisms of recent selection.

Distinguishing between the different mechanisms of balancing selection acting upon classical MHC-I alleles is far from trivial and is beyond the scope of the present study. Both heterozygote advantage and negative frequency-dependent selection can potentially disfavour common alleles, and both mechanisms may be operating in tandem, along with fluctuating selection [5]. It has been suggested that balancing selection is more common in adaptive evolution than previously thought [33], probably mediated by changes in the level of allele or gene expression [34]. Moreover, PMBS acts on expressed alleles, and, under negative frequency-dependent selection, rare alleles can only increase the host's fitness if they are expressed [33]. Our findings, in house sparrows, reveal that the level of expression of classical MHC-I alleles varies considerably within an individual's genotype and highly and lowly expressed alleles have been subject to different magnitudes of balancing selection.

Consistent with earlier findings [27], we found that house sparrows are likely to have at least two expressed classical and two expressed non-classical MHC-I genes. Among the expressed classical alleles, each individual possessed one highly expressed classical MHC-I allele and one moderately expressed classical allele. Given that high heterozygosity is expected for classical MHC genes, the two alleles exhibiting moderate-to-high expression probably belong to one gene. The majority of individuals also had one or two lowly expressed classical alleles, which probably equate to one lowly expressed classical gene. These findings are comparable to the ‘major’ and ‘minor’ classical MHC genes reported in the domestic chicken *Gallus gallus*, where each individual has a *‘*major’ MHC gene, which is highly expressed and encodes an MHC protein whose peptide-binding specificity determines the immune response to key pathogens, while a ‘minor’ gene is expressed at a lower level [19,35,36]. The single highly expressed classical MHC-I gene that we observe in the house sparrow could be considered to be on a par with the chicken's ‘major’ MHC gene, while the lowly expressed classical MHC-I alleles belong to genes akin to the ‘minor’ MHC gene in the chicken. It would thus be expected that the ‘major’ house sparrow MHC-I locus is subject to stronger balancing selection than the ‘minor’ locus; however, since we are not yet able to sort MHC-I alleles by locus, in the house sparrow, this theory remains to be proven. As in the chicken, the number of expressed classical genes varies among individuals in house sparrows, but the putative ‘major’ gene is always present and expressed.

Despite having a larger number of non-classical MHC-I alleles in genomic DNA, house sparrows did not express more non-classical alleles, compared with classical alleles, and none of the non-classical alleles were expressed at a high level. In blood we found that, in the average sparrow, 57% of the classical alleles were expressed while 38% of the non-classical alleles were expressed. While we cannot exclude the fact that expression patterns might be different in other tissues, we have unpublished data suggesting that the expression patterns of classical MHC-I alleles are highly similar across tissues in the house sparrow. Our findings are consistent with a previous study in house sparrows [27] confirming that only a proportion of MHC-I alleles are expressed and the number of expressed non-classical alleles is disproportionately lower than the number of expressed classical alleles. Hence, the expressed MHC diversity in blood is considerably lower than the genomic MHC diversity. Future studies should seek to compare expression patterns across tissues to confirm that blood is representative of the system-wide response and is suitable for studying selection at the MHC-I region.

In conclusion, it has been long understood that there are two types of MHC-I genes, classical and non-classical, exhibiting different characteristics in terms of polymorphism and function. Our results indicate recent selection at the MHC in a natural population, as inferred by allele frequencies, which supports studies of historic selection by suggesting that classical and non-classical MHC-I genes are subject to opposing selection pressures. Importantly, we also provide evidence for a further distinction, in which classical MHC-I alleles can be separated by frequency and expression into the rare highly or moderately expressed classical alleles subject to strong PMBS and the common lowly expressed classical alleles subject to weak PMBS. It is these rare, moderately-to-highly expressed classical MHC-I alleles that are likely to play a central role in ongoing host–pathogen co-evolution, whereas the common lowly expressed classical alleles may have lost their advantage and instead be selected against. While we did not correlate MHC-I expression profiles with fitness-related traits, such as reproductive success or disease resistance, the strengths of our approach are that (i) we have *a priori* predictions for the expression profiles of the two gene types, (ii) we know that MHC-I alleles are inherited in linked haplotypes [18] and (iii) our preliminary genome assembly places the classical and non-classical genes close together (H. Westerdahl, unpublished data). We predict that a similar pattern exists in other songbirds; even though songbirds exhibit high duplication of the MHC-I genes, only a few genes may be highly expressed and subject to strong PMBS at any given point in time.

## 4. Methods

### (a) Study system, sites and sampling design

The house sparrow is one of the most widely distributed terrestrial birds. With a native range in Europe and Central Asia, in the last two centuries, the house sparrow—aided by humans—has successfully invaded all continents, except Antarctica. Its invasion success has partly been attributed to immune defence strategies [37]. The species is highly sedentary with limited natal dispersal. House sparrows were captured in four countries in Europe, spanning a latitudinal gradient of 17 degrees. Sampling locations were in Spain (38.832064°, −6.916733°), Bulgaria (44.011582°, 26.439325°), Poland (51.242974°, 16.932992°) and Sweden (55.737356°, 13.62987°) (for further details, see [38]). Birds were captured in September 2016 and May 2017, and blood was collected, along with data on sex, age and biometrics. In total, 28 birds were sampled (*n* = 7 per country, approximately 50 : 50 between spring and autumn). No individuals were captured in more than one sampling season. Animal procedures were approved by the relevant regulatory authorities (see Ethics statement).

### (b) Nucleic acid extraction, library preparation and sequencing of MHC-I

gDNA was extracted from whole blood stored in SET buffer by ammonium acetate [39]. RNA was extracted from whole blood stored in RNA*later* (Qiagen) following a protocol combining TRIzol LS (Thermo Fisher Scientific) and Qiagen's RNeasy Plus Mini kit. Firstly, to separate blood from RNA*later*, samples were spun at 20 000*g* for 1 min, following which the supernatant was discarded. The blood pellet was mixed with 250 µl of 1 × PBS, followed by lysis and phase-separation according to the Trizol LS manufacturer's protocol. This yielded an aqueous phase that was added to the gDNA Eliminator Mini Spin Columns and, from hereon, the Qiagen RNeasy Plus Mini kit manufacturer's protocol was followed. Extracted RNA was reverse transcribed to produce cDNA using SuperScript IV Reverse Transcriptase (Thermo Fisher Scientific) with oligo (dT) following the manufacturer's protocol.

Library preparation proceeded in the same way for both gDNA and cDNA, although with slightly different forward primers. Exon 3 of MHC-I was amplified by PCR using validated primers with an overhang for attachment of index tags (see below). The primers used for gDNA amplification were: HNalla (forward primer; 5′–TCCCCACAGGTCTCCACAC–3′) [40] and Spsp–r2 (reverse primer; 5′–TTGCGCTCCAGCTCCYTCT–3′) [26]. cDNA was amplified using the forward primer exon2_fwd1 (5′–GAGCGGGGGTCTCCACAC–3′) and the same reverse primer as for gDNA. Forward primers for both gDNA and cDNA were placed at the same position, such that both primer combinations cover the same region of exon 3; the difference is that the gDNA forward primer starts in the intron, while the cDNA forward primer starts in exon 2. A 25 µl reaction volume contained 25 ng gDNA, 0.5 µM of forward and reverse primers (Eurofins Genomics), 12.5 µl of 2X Phusion Master Mix with HB Buffer (Thermo Fisher Scientific) and ddH_2_O. PCR settings for gDNA amplification were 30 s at 98°, followed by 28 cycles of 10 s at 98°C, 10 s at 66°C and 10 s at 72°C, then 10 min at 72°C and cooling to 4°C (GeneAmp PCR System 9700, Applied Biosystems). PCR settings for cDNA amplification were 30 s at 98°, followed by 25 cycles of 10 s at 98°C, 10 s at 66.5°C and 6 s at 72°C, then 10 min at 72°C and cooling to 4°C.

Samples were cleaned using Agencourt AMPure XP-PCR purification beads (Beckman Coulter) with minor modifications to the manufacturer's protocol: beads were used at a ratio of 0.8 relative to PCR product; beads were cleaned with 80% EtOH; and samples were eluted in 43 µl ddH_2_O with an incubation of 2 min at room temperature. Cleaned PCR products were verified by gel electrophoresis. Dual-indexing primers (Nextera XT Index Kit v2, Illumina) were attached in a second PCR to uniquely index each sample. A 50 µl reaction volume contained 25 µl 2X Phusion Master Mix, 5 µl each of the two index primers, 5–15 µl cleaned PCR product (volume dependent on the strength of the amplification as determined by evaluation of bands on agarose gel) and ddH_2_O to make up to 50 µl. PCR settings were 30 s at 98°C, followed by 8 cycles of 10 s at 98°C, 15 s at 62°C and 15 s at 72°C, followed by 10 min at 72°C and cooling to 4°C. PCR products were cleaned as described above using beads at a ratio of 1.12 relative to the PCR product.

DNA concentration of the final cleaned PCR products was quantified using the Quant-iT PicoGreen dsDNA Assay Kit (Invitrogen), as per the manufacturer's instructions with minor modifications for 96-well microplates: 2 µl PCR product was diluted with 98 µl 1 × TB and 100 µl PicoGreen was added. Fluorescence was quantified using a FLUOstar Omega plate reader (BMG Labtech) and blank-corrected, and concentrations were calculated relative to a standard curve run in duplicate ranging from 7.8^e−3^ to 0.5 ng µl^−1^. Samples were pooled to give equimolar DNA per sample. Paired-end sequencing (300 bp) was performed on the Illumina MiSeq platform at the DNA Sequencing Facility at Lund University, Sweden. A total of 11% and 43% of samples were included as duplicates in gDNA and cDNA sequencing, respectively.

### (c) Processing of sequence data

Raw reads were processed to remove adapters and cut to the expected length (245 bp) using Cutadapt v. 1.14 [41]. Read quality was assessed using FastQC v. 0.11.7 [42] and MultiQC v. 1.4 [43]. Trimmed reads were processed with DADA2 v. 1.10.0 [44] in R 4.1.0 [45], using the following settings: gDNA: truncLen = 214, 190; trimLeft = 0, 10; maxN = 0; maxEE = 2, 2; and cDNA: truncLen = 230, 220; maxN = 0; maxEE = 2, 3; where truncLen is the length at which reads are truncated, trimLeft indicates the number of base pairs to be trimmed at the start of reads, maxN is the number of unresolved bases allowed and maxEE is the maximum expected number of errors allowed; where two values are given, these refer to parameter settings for forward and reverse reads, respectively. Note that 10 bp was trimmed from the start of reverse gDNA reads due to poor quality. Further filtering removed sequences that fell below a minimum threshold frequency of 2.1% and 0.25% for gDNA and cDNA, respectively. Optimal thresholds for read truncation and maxEE were determined by assessment of mismatches among replicated samples (gDNA: *n* = 17; cDNA = 12) to achieve the highest possible repeatability while losing minimal data. Settings were evaluated over the following ranges: gDNA: truncation of forward reads = 220–240; truncation of reverse reads = 180–200; maxEE of 2, 2 and 2, 3; cDNA: truncation of forward reads = 230–240; truncation of reverse reads = 210–220; maxEE of 2, 2 and 2, 3. Average (mean ± s.e.m.) read depths per sample were 6866.3 ± 305.3 in gDNA and 26973.4 ± 773.03 in cDNA. Where replicates differed, the replicate with the highest number of alleles was taken forward into downstream processing and analysis. Nucleotide sequences were translated into amino acid sequences using Aliview [46]. To ensure matching lengths between gDNA and cDNA, 10 bp was cut from the start of cDNA sequences, which were subsequently matched to corresponding gDNA alleles using seqeqseq and default parameters (http://130.235.244.92/apps/seqeqseq.html).

### (d) Data analysis

All further data processing and analysis were performed in R 4.1.0. Alleles were classified as classical or non-classical, with non-classical alleles identified as having a 6 bp deletion [18,27]. Filtered read counts were converted into allele presence/absence for gDNA and cDNA, at the levels of nucleotide and amino acid, and the numbers of unique gDNA and cDNA alleles were summed for each individual. Generalized linear models (GLMs) were fitted using the glm function in the stats package, while generalized linear mixed models (GLMMs) were fitted using the glmmTMB package in which models are fitted using maximum-likelihood estimation via the template model builder (TMB). All analyses were performed for both nucleotide and amino acid alleles; results from nucleotide alleles are in the main text, while results from amino acid alleles can be found in the electronic supplementary material.

### (e) Genomic and expressed MHC-I diversity

Firstly, we fitted GLMMs with Poisson errors to the total number of (*a*) genomic and (*b*) expressed alleles per individual including the fixed effect of allele type (classical or non-classical) to characterize MHC diversity. The model fitted in (*b*) enabled us to test (i) the prediction that expressed diversity is lower than genomic diversity by including the fixed effect of number of genomic MHC-I alleles and (ii) if expressed diversity is disproportionate between classical and non-classical alleles, by including the interaction between number of genomic alleles and allele type. In both (*a*) and (*b*), we also considered the need to control for temporal variation in MHC diversity (e.g. if sampling different subsets of the population) by including the fixed effect of season (two-level factor: spring or autumn). A random effect of individual identity nested in sampling site to control for non-independence associated with repeated measures from individuals (measures for both classical and non-classical alleles) and sampling sites.

### (f) MHC-I genomic allele frequencies

To test the hypothesis that more classical MHC-I alleles are rare, while non-classical alleles are more frequently shared among individuals, we first binned genomic alleles (separated into classical and non-classical alleles) according to the number of individuals in which they were found, deriving an allele count for each observation frequency (i.e. number of individuals). A GLM with Poisson errors was fitted to allele counts, including the fixed effects of allele frequency (number of individuals in the population having an allele), allele type and their interaction.

### (g) Phylogenetic analysis

To determine the phylogenetic relationship between MHC-I alleles, a model selection test was run in MEGA X [47] using a Kimura two-parameter model, with a gamma distribution and 500 bootstrap replications. A maximum-likelihood tree was constructed using iTOL v. 6 [48].

### (h) MHC-I expression in relation to allele frequency

To test the hypothesis that rare classical alleles are more likely to be expressed and expressed at higher levels, compared with common classical alleles, while expression of non-classical alleles is unrelated to allele frequency, we analysed variation in the probability and level of expression in relation to allele type and frequency. Having confirmed overdispersion in the data, we fitted two-part zero-altered negative binomial mixed models (family = *nbinom2*) to allele read counts, enabling us to simultaneously model the probability of expression of MHC-I alleles (0 or 1; zero count model) and the level of expression of the expressed MHC-I alleles (conditional count model). The zero-count model included allele type, allele frequency, total number of genomic alleles, and the interaction between allele type and allele frequency, while the conditional count (non-zero) model included the fixed effects of allele type, allele frequency, cDNA read depth (log-transformed), total number of expressed alleles, and the interaction between allele type and allele frequency. Again, we considered the need to account for potential variation due to sampling season by including the variable in saturated models for both the zero counts and non-zero counts. A random effect of individual identity nested in sampling site was included in both parts of the model. A random effect of allele identity was not included due to a strong correlation between allele frequency and type. Since there is the possibility that primer specificity could lead to biased representations of expression level (by inflating read counts), we first checked for a correlation between allele read counts in cDNA and gDNA, using Pearson's correlation test.

### (i) Expression within the MHC-I genotype

To investigate variation in expression levels within an individual's expressed MHC genotype, each individual's expressed alleles were first ranked in order of decreasing expression. A linear mixed model (LMM) with Gaussian errors was fitted to square-root transformed cDNA read counts (no zeroes) including the fixed effects of rank (five-level ordered factor), allele type, cDNA read depth (log-transformed), total number of expressed alleles, the interaction between rank and allele type and a random effect of individual identity. Since rank was defined as an ordered factor, we employed polynomial regression, which fits polynomial contrasts successively, with increasing degrees (i.e. linear, quadratic, cubic, etc.) until *p* > 0.05. Due to model convergence issues and very low variance associated with the nested random effect of site/individual, site was not included in the random effects structure. To confirm that expression levels are not influenced by primer specificity and amplification bias, a similarly structured LMM with Gaussian errors was fitted to square-root transformed gDNA read counts, including the fixed effects of rank (five-level ordered factor), allele type, gDNA read depth (log-transformed), total number of genomic alleles, the interaction between rank and allele type and a random effect of individual identity.

### (j) Model selection and evaluation for linear mixed models and generalized linear mixed models

A model selection process was performed for each analysis. For all models, terms were eliminated one-by-one from models if they did not significantly improve the model fit when comparing nested models with likelihood ratio chi-square tests (GLM and GLMM) or Wald chi-square tests (zero-altered negative binomial). Since the latter had to be performed separately on the zero-count and conditional-count parts of the model, additional validation was carried out using Akaike's information criterion to compare the full and reduced models. Minimum adequate models were evaluated by inspection of residuals. For zero-altered binomial models, diagnostics were performed using the DHARMa package. Estimated marginal (EM) means (factors) and EM trends (covariates) and their 95% confidence intervals (CI) were derived for significant interactions, and interaction contrasts were assessed using the emmeans package with *p*-value adjustment using the Tukey method.

### (k) Estimating selection on MHC-I

To estimate selection on MHC-I genes, we employed several methods to identify sites under positive selection in genomic and expressed MHC-I alleles, separating the classical and non-classical alleles. Expressed classical alleles were further separated into highly expressed (rank 1), moderately expressed (rank 2) and lowly expressed (ranks 3–5) alleles. Selection analyses aimed to test two hypotheses: (i) expressed classical alleles have more sites under positive selection compared with expressed non-classical alleles, and (ii) among expressed classical alleles, those that are highly expressed have more sites under positive selection compared with those that are lowly expressed. Firstly, codon trees were built in IQ-TREE v. 1.6.1 [49], and the best-fit substitution model was determined [50] based on one alignment (including full codons) for each set of alleles, as described above. Codon tests of positive selection were performed on aligned sequences using codeml in PAML [51], fitting five models of codon evolution: M1a (neutral), M2a (selection), M7 (beta), M8 (beta&*ω*) and M8a (beta&*ω*s = 1). Nested models were compared by log-likelihood ratio tests to identify the best-fit model (M1a versus M2a, M7 versus M8 and M8a versus M8), and positively selected sites were estimated using Bayes empirical Bayes method [52] at a threshold significance of 0.05. Evidence for recombination was first tested using genetic algorithm for recombination detection (GARD) [53] using Hyphy, confirming that there was no evidence for recombination in either genomic or expressed alleles separated into each of the classical and non-classical alleles.

## Data Availability

Raw read data are deposited in NCBI's Sequence Read Archive under the BioProject PRJNA1064702 [54]. New nucleotide sequences for the putative alleles described in this study are deposited in GenBank (accession nos.: PP114378–PP114491). Supporting data and code are available from the Dryad Digital Repository [55]. Supporting analyses are provided in electronic supplementary material [56].
